# Current update on the neurological manifestations of long COVID: more questions than answers

**DOI:** 10.17179/excli2024-7885

**Published:** 2024-11-27

**Authors:** Maria-Ioanna Stefanou, Evangelos Panagiotopoulos, Lina Palaiodimou, Eleni Bakola, Nikolaos Smyrnis, Marianna Papadopoulou, Christos Moschovos, George P. Paraskevas, Emmanouil Rizos, Eleni Boutati, Elias Tzavellas, Stylianos Gatzonis, Annerose Mengel, Sotirios Giannopoulos, Sotirios Tsiodras, Vasilios K. Kimiskidis, Georgios Tsivgoulis

**Affiliations:** 1 Second Department of Neurology, National and Kapodistrian University of Athens, School of Medicine, “Attikon” University Hospital, Athens, Greece; 2 Department of Neurology & Stroke, Eberhard-Karls University of Tuebingen, Tuebingen, Germany; 3 Hertie Institute for Clinical Brain Research, Eberhard-Karls University of Tuebingen, Tuebingen, Germany; 4 Second Department of Psychiatry, National and Kapodistrian University of Athens, School of Medicine, "Attikon" University Hospital, Athens, Greece; 5 Department of Physiotherapy, University of West Attica, Athens, Greece; 6 Second Propaedeutic Department of Internal Medicine and Research Institute, University General Hospital Attikon, National and Kapodistrian University of Athens, Athens, Greece; 7 First Department of Psychiatry, "Aiginition" Hospital, School of Medicine, National and Kapodistrian University of Athens, Athens, Greece; 8 Department of Neurosurgery, National and Kapodistrian University of Athens, Athens, Greece; 9 Fourth Department of Internal Medicine, Attikon University Hospital, National and Kapodistrian University of Athens, School of Medicine, Athens, Greece; 10 First Department of Neurology, AHEPA University Hospital, Aristotle University of Thessaloniki, Thessaloniki, Greece

**Keywords:** long COVID, long-haul, PACS, COVID-19, brain fog, SARS-CoV-2

## Abstract

Since the outbreak of the COVID-19 pandemic, there has been a global surge in patients presenting with prolonged or late-onset debilitating sequelae of SARS-CoV-2 infection, colloquially termed long COVID. This narrative review provides an updated synthesis of the latest evidence on the neurological manifestations of long COVID, discussing its clinical phenotypes, underlying pathophysiology, while also presenting the current state of diagnostic and therapeutic approaches. Approximately one-third of COVID-19 survivors experience prolonged neurological sequelae that persist for at least 12-months post-infection, adversely affecting patients' quality of life. Core neurological manifestations comprise fatigue, post-exertional malaise, cognitive impairment, headache, lightheadedness ('brain fog'), sleep disturbances, taste or smell disorders, dysautonomia, anxiety, and depression. Some of these features overlap substantially with those reported in post-intensive-care syndrome, myalgic encephalomyelitis/chronic fatigue syndrome, fibromyalgia, and postural-orthostatic-tachycardia syndrome. Advances in data-driven research utilizing electronic-health-records combined with machine learning and artificial intelligence have propelled the identification of long COVID sub-phenotypes. Furthermore, the evolving definitions reflect the dynamic conceptualization of long COVID in both research and clinical contexts. Although the underlying pathophysiology remains incompletely elucidated, neuroinflammatory responses, endotheliopathy, and metabolic imbalances, rather than direct viral neuroinvasion, are implicated in neurological sequelae. Genetic susceptibility has also emerged as a potential risk factor. While major limitations remain with existing definitions, collaborative strategies to standardize diagnostic approaches are needed. Current therapeutic paradigms advocate for multimodal approaches, integrating pharmacological and non-pharmacological interventions along with comprehensive rehabilitation programs. Although preliminary evidence of therapeutic efficacy has been provided by a number of clinical trials, methodological constraints limit the generalizability of this evidence. Preventive measures, notably vaccination, have proven integral for reducing the global burden of long COVID. Considering the healthcare and socioeconomic repercussions incurred by long COVID worldwide, international collaborative initiatives are warranted to address the remaining challenges in diagnosing and managing patients presenting with neurological sequelae.

See also the graphical abstract(Fig. 1).

## Introduction

The global impact of the Coronavirus Disease 2019 (COVID-19) pandemic, caused by the severe acute respiratory syndrome coronavirus 2 (SARS-CoV-2), has been profound, resulting in significant mortality and morbidity worldwide, with ramifications extending to prolonged or late-onset debilitating sequelae of SARS-CoV-2 infection, colloquially termed “long COVID”. At present, the death toll of the COVID-19 pandemic has exceeded 7 million worldwide (Ely et al., 2024[30]). By comparison, it is estimated that the global cases of long COVID surpass 65 million, based on conservative estimates of a 10 % incidence among the more than 651 million documented COVID-19 cases globally (Davis et al., 2023[24]). The incidence of long COVID has also been reported to be twofold higher among hospitalized compared to non-hospitalized patients, with incidence estimates ranging between 50-70 % and 10-30 %, respectively, while being less frequent among vaccinated individuals, occurring in 10-12 % of cases (Al-Aly et al., 2022[2]; Davis et al., 2023[24]).

Currently, long COVID is conceptualized as a multisystem post-infectious disorder; yet, the breadth of clinical symptoms and phenotypes reported by long COVID patients has posed significant challenges in devising and implementing reliable definitions for clinical practice. More than 200 symptoms have been ascribed to long COVID to date, with significant overlap observed between long COVID and other conditions, including myalgic encephalomyelitis/chronic fatigue syndrome (ME/CFS), fibromyalgia (FM), post-intensive care syndrome (PICS), and postural orthostatic tachycardia syndrome (POTS) (Davis et al., 2021[23]; Goldenberg, 2024[35]). 

While the diagnostic boundaries of long COVID remain far from sharply defined, significant advances have been made in the past few years. With the accrual of epidemiological studies, the clinical evidence regarding long COVID has begun to consolidate, indicating that fatigue, post-exertional malaise, concentration and memory difficulties, recurring headaches, lightheadedness (often referred to as 'brain fog'), shortness of breath, cough, sleep disturbances, taste or smell disorders, and gastrointestinal symptoms are among the most frequently reported (Ely et al., 2024[30]). The growing body of epidemiological data has also prompted a reappraisal of long COVID, as reflected by the gradual shift in its definitions-from those initially introduced by the CDC (Centers for Disease Control and Prevention) and NICE (National Institute for Health and Care Excellence), to those later proposed by the WHO (World Health Organization) and more recently by NASEM (National Academies of Sciences, Engineering, and Medicine) (Table 1(Tab. 1)) (Chou et al., 2024[20]; Ely et al., 2024[30]; Soriano et al., 2022[78]). Although criticism has been raised that a robust understanding of long COVID is still distant as long as its pathophysiology and validated biomarkers remain elusive, these systematic attempts to refine its definition highlight the importance of deploying an evidence-based and standardized diagnostic framework as the first step in the clinical approach to managing long COVID patients.

In this context, neurological manifestations are among the most prevalent and disabling sequelae of long COVID, leading to significant impairment in patients' quality of life and posing substantial challenges for healthcare systems worldwide. Fatigue and sleep disturbances, along with cognitive impairment, are particularly common, with reported prevalence rates ranging from 20 % to 60 % and 20 % to over 50 %, respectively (Ahmed et al., 2020[1]; Raman et al., 2021[71]). Psychiatric complications, including anxiety and depression, are also frequently observed, affecting 20 % to 40 % of long COVID patients, while headache is recorded in approximately 10 % to 20 % of cases (Efstathiou et al., 2022[27][28][29]; Mazza et al., 2020[50]; Membrilla et al., 2021[52]). Other neurological symptoms, such as dizziness and balance disorders, are experienced by approximately 10 % to 30 %, while neuropathic pain is documented in 10 % to 20 % of cases (Malik et al., 2022[48]; Taquet et al., 2021[87]). Additional neurological symptoms reported by long COVID sufferers include dysautonomia, taste or smell impairment, tinnitus, and neuromuscular symptoms, such as paraesthesias, muscle weakness, myalgia, twitching, and myoclonic jerks (Davis et al., 2021[23]; Spinato et al., 2020[79]; Tsivgoulis et al., 2020[91], 2021[92]). Collectively, the neurological manifestations of long COVID are linked to significant reductions in work capacity and a substantial and, thus far, incremental socioeconomic burden worldwide (Wolff Sagy et al., 2023[97]).

Our collaborative group has previously addressed the prevalence, key components, and pathophysiological mechanisms of long COVID (Stefanou et al., 2022[82]). We currently attempt to provide an updated and comprehensive overview of this condition, analyzing key recent advances in unraveling its underlying pathophysiology as well as potential diagnostic and treatment implications. Additionally, we propose a standardized framework for the clinical approach and management of patients presenting with neurological manifestations of long COVID, along with directions for future research.

## Neurological Aspects of Long COVID: Prevalence, Risk Factors, and Temporal Dynamics

In clinical practice, it is apparent that the neurological manifestations of long COVID, as well as those affecting other organ systems, are intertwined, rendering them primarily discernible for classification purposes. A significant concern for clinicians is that non-specific symptoms-such as fatigue, difficulties with concentration or memory, 'brain fog,' post-exertional malaise, and sleep disturbances-may represent secondary manifestations of underlying conditions affecting the respiratory, cardiovascular, endocrine, renal, hematologic, gastrointestinal, autoimmune, or psychiatric systems. Consequently, a meticulous and, in most cases, interdisciplinary assessment is imperative to prevent the erroneous attribution of these neurological symptoms to long COVID, when they may in fact originate from other underlying disorders that comprise long COVID mimics (Stefanou et al., 2022[82]).

Recent meta-analyses have provided detailed prevalence estimates for various neurological manifestations of long COVID. One such meta-analysis included 36 studies with a total sample size of 11,598 long COVID patients (Natarajan et al., 2023[55]). Fatigue emerged as the most frequently reported neurological symptom, documented in 29.2 % of long COVID cases. Cognitive impairment was also notably prevalent, with a prevalence rate of approximately 28.8 %, followed by attention/concentration and memory deficits at 20.2 % and 18.4 %, respectively. Psychiatric symptoms, including depression, anxiety, posttraumatic stress disorder (PTSD), and sleep disturbances, were also frequent among COVID survivors, with a pooled prevalence of 21.3 %. Impairments of smell and taste were significant, with rates around 14.8 %. Hearing disturbance, headache and dizziness were reported with pooled prevalence rates of 12.9 %, 10.5 % and 9.1 %, respectively. These estimates were characterized by considerable heterogeneity and broad confidence intervals, highlighting the significant variability among studies (Natarajan et al., 2023[55]). These findings emphasize the extensive and varied neurological manifestations observed in long COVID populations but also point to the methodological discrepancies present in the currently published epidemiological studies.

In line with the previous evidence, a larger meta-analysis comprising 194 studies and totaling 735,006 participants disclosed similar results, indicating that fatigue ranks among the most prevalent long COVID symptoms, affecting nearly one-third of COVID-19 survivors regardless of hospitalization status (28.4 % in hospitalized, 34.8 % in non-hospitalized, and 25.2 % in mixed cohorts) (O'Mahoney et al., 2023[58]). It should be noted, however, that contradictory data also exist in the literature, with some studies identifying hospitalization as an independent risk factor for long COVID (Ayoubkhani et al., 2021[5]); a finding that has been attributed to the significant overlap noted between long COVID and PICS. Remarkably, the pooled prevalence estimates of this study align with those from other meta-analyses, indicating that among neurological symptoms, generalized pain/ discomfort (27.9 %), sleep disturbances (23.4 %), cognitive impairments and memory deficits (19.9 %), concentration difficulties (18.6 %), PTSD (16.5 %), anxiety and depression (13.9 %), myalgias (10.3 %), and headache (6.4 %) are the most frequently reported (Efstathiou et al., 2022[27]; O'Mahoney et al., 2023[58]). Additionally, disturbances in taste/ smell, paresthesias, and dizziness were documented in 6.3 %, 6.2 %, and 6.2 % of patients, respectively. Although an exhaustive account of neurological symptoms reported by long COVID sufferers is neither possible nor meaningful in the context of the present review, it is important to note that with the accrual of data, the neurological manifestations of long COVID are increasingly distinguished from other nosological entities. Efforts based on data-driven analyses of electronic health records (EHRs), employing machine learning and artificial intelligence, are also expected to expedite the characterization of long COVID sub-phenotypes and further refine current definitions (Dagliati et al., 2023[21]; Zang et al., 2024[101]).

In terms of prognostic risk factors, though no consensus has yet been established in the literature, a large-scale analysis of EHRs involving 4,676,390 participants indicated that compared to the general population, individuals with long COVID are more likely to be females (65.1 % vs. 50.4 %), fall within the age range of 38-67 years (63.7 % vs. 48.9 %), be overweight or obese (45.7 % vs. 29.4 %), and have at least one comorbidity (52.7 % vs. 36.0 %) (Jeffrey et al., 2024[42]). Additionally, these individuals are more likely to have contracted the virus before the Omicron variant became dominant (44.9 % vs. 35.9 %). Importantly, in a number of large well-designed real-world studies, vaccination with any COVID-19 vaccine has been linked to a 50 % reduction of the risk of developing long COVID (Català et al., 2024[16]; Trinh et al., 2024[90]). 

It is crucial to note that COVID-19 vaccines have also been associated with a wide spectrum of neurological adverse events, ranging from mild neurological symptoms such as headache, dizziness, and transient paresthesia, to more severe complications, including cerebral venous sinus thrombosis, acute ischemic stroke, Guillain-Barré syndrome, transverse myelitis, acute disseminated encephalomyelitis, Bell's palsy, and exacerbation of underlying neurological disorders (Beatty et al., 2021[9]; Palaiodimou et al., 2021[60], 2022[59]; Papadopoulou et al., 2023[61][63]; Stefanou et al., 2022[80][82]). Additionally, complications affecting other organ systems, such as allergic reactions, anaphylaxis, and myocarditis, have been reported (Buoninfante et al., 2024[12]). While certain idiosyncratic, genetic, and environmental factors may predispose individuals to neurological adverse events, real-world data emphasize the rarity of serious adverse effects among patients exposed to various types of COVID-19 vaccines (Beatty et al., 2021[9]). Indeed, when weighed against the risks of SARS-CoV-2-related complications, including long COVID, the existing literature demonstrates a clear net benefit of vaccination at the population level (Català et al., 2024[16]; Stefanou et al., 2022[81], 2023[83]).

Regarding the temporal trends of long COVID, it is important to note that outcomes arising from a mild disease course typically tend to subside or return to the premorbid baseline within the first year for the majority of patients (Table 2(Tab. 2); References in Table 2: Arnold et al., 2021[4]; Badenoch et al., 2022[6]; Dorobisz et al., 2023[25]; Li et al., 2023[46]; Păunescu et al., 2023[64]; Peter et al., 2022[66]; Rivera-Izquierdo et al., 2022[73]; Tana et al., 2022[86]; Vaira et al., 2020[93]) (Mizrahi et al., 2023[53]). Nevertheless, research indicates a significant variability in the prevalence and duration of neurological long COVID symptoms. For instance, while symptoms comprising smell and taste disorders tend to improve markedly within the first weeks to months from contraction, fatigue is reported by approximately 58 % of patients for several months post-COVID-19 infection (Davis et al., 2021[23]). In a recent meta-analysis that included 742 studies with long-term follow-up data from 7,912 COVID-19 survivors, the most prevalent long COVID symptoms two years following SARS-CoV-2 infection were fatigue (28.0 %), cognitive impairment (27.6 %), anxiety (13.4 %), depression (18.0 %), sleep disturbances (20.9 %), and pain (8.4 %) (Fernandez-de-Las-Peñas et al., 2024[31]). Importantly, a recent meta-analysis that provided very robust data regarding neurological manifestations of long COVID, encompassing data from 63 controlled cohort studies with more than 96 million participants, indicated that although clusters of non-neurological long COVID symptoms tend to subside markedly within 12 months from SARS-CoV-2 infection (i.e., including urological, cardiovascular, and gastrointestinal symptoms), there was a significantly increased risk for persisting neurological symptoms at 12-month follow-up in infected individuals versus controls (risk ratio: 1.51; 95 % confidence interval: 1.17 to 1.93) (Franco et al., 2024[32]). These findings underscore the complex and highly variable nature of long COVID, with a significant proportion of patients experiencing protracted recovery periods and long-term neurological sequalae.

## Pathophysiological Underpinnings of Neurological Manifestations of Long COVID

**Figure 2:**
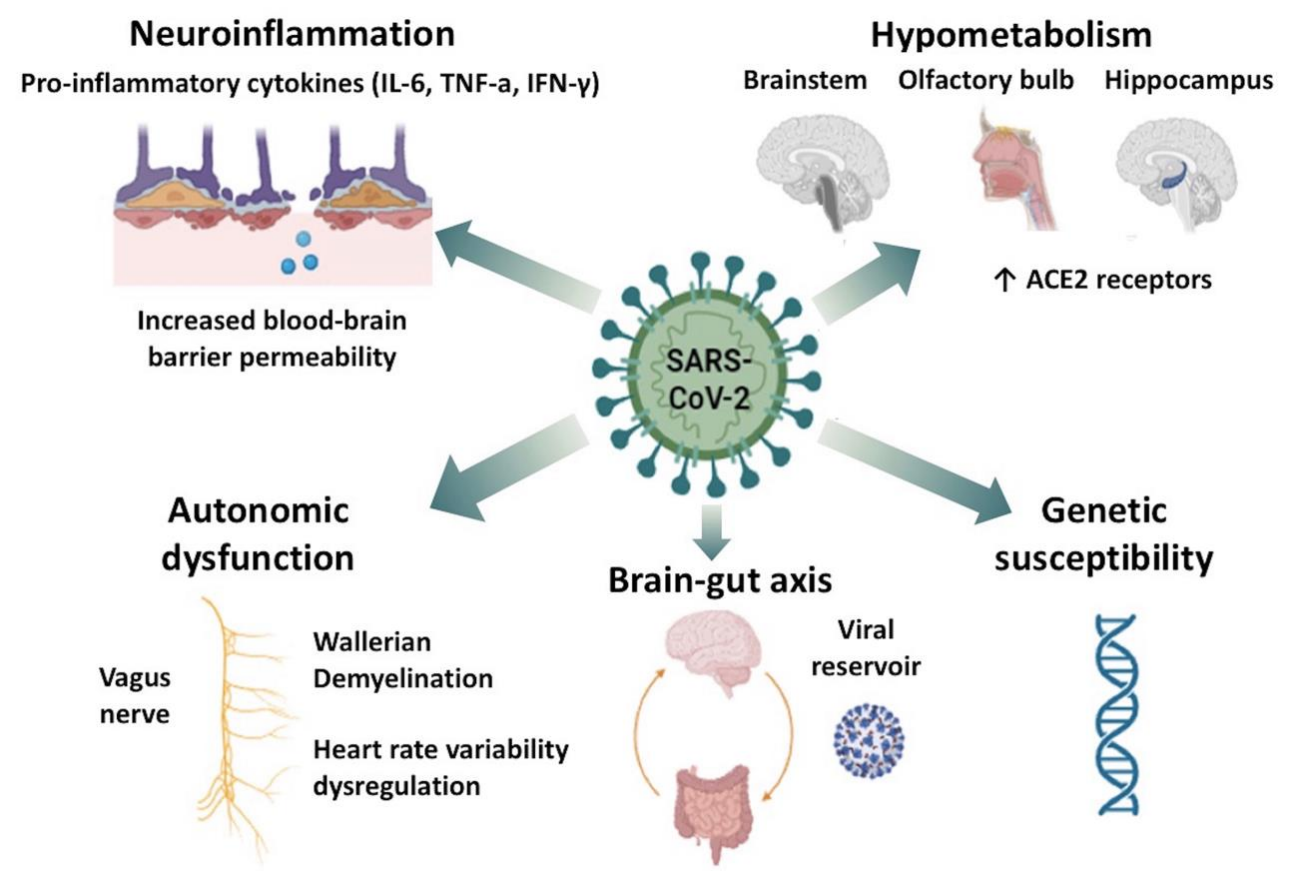
Pathophysiological mechanisms implicated in neurological manifestations of long COVID. The pathophysiology of long COVID is multifactorial, with several mechanisms contributing to its neurological manifestations. Neuroinflammation is a key feature, driven by persistent immune activation involving elevated levels of pro-inflammatory cytokines and autoantibodies, leading to chronic inflammation in both the central and peripheral nervous systems. Reactivation of latent viruses, such as Epstein-Barr virus, may further exacerbate these inflammatory processes. Endothelial dysfunction plays a critical role, characterized by damage to the blood-brain barrier (BBB), which increases its permeability, allowing inflammatory mediators and immune cells to infiltrate the CNS, potentially leading to cognitive deficits such as brain fog. Metabolic disturbances are observed in regions with a high density of ACE2 receptors, such as the olfactory bulb, hippocampus, and brainstem, where hypometabolism likely results from mitochondrial dysfunction and oxidative stress, disrupting cognitive function, mood regulation, and autonomic processes. Autonomic dysfunction is another significant component, marked by dysregulation of autonomic nervous system functions, including impaired heart rate variability, possibly due to damage to the brainstem and vagus nerve. The viral reservoir hypothesis posits that while direct viral persistence in the CNS is rare, SARS-CoV-2 reservoirs in peripheral organs, such as the lungs and gastrointestinal tract, may perpetuate systemic inflammation, indirectly affecting both the CNS and PNS. This inflammation can disrupt the brain-gut axis, potentially exacerbating neurological symptoms by influencing neuroinflammation and gut-derived inflammatory mediators. Finally, genetic susceptibility may influence the risk of developing long COVID, with variants in immune-regulatory genes such as OAS1 and TLR4 potentially contributing to prolonged immune responses and viral persistence, though the exact genetic determinants remain to be fully elucidated. This image was created with BioRender (https://biorender.com). ACE2: Angiotensin-Converting Enzyme 2; BBB: Blood-Brain Barrier; CNS: Central Nervous System; COVID: Coronavirus Disease; EBV: Epstein-Barr Virus; PNS: Peripheral Nervous System; SARS-CoV-2: Severe Acute Respiratory Syndrome Coronavirus 2; TLR4: Toll-Like Receptor 4; OAS1: 2'-5'-Oligoadenylate Synthetase 1

The pathophysiology underlying the neurological manifestations of long COVID is complex and multifactorial, involving both direct and indirect effects of SARS-CoV-2 on the central nervous system (CNS) and peripheral nervous system (PNS) (Figure 2(Fig. 2)). Despite the extensive research on long COVID in the past few years, the exact mechanisms still remain incompletely elucidated. Today, there is converging evidence suggesting that the long-term neurological sequelae are primarily driven by sustained neuroinflammatory responses, endothelial dysfunction, and metabolic disturbances, rather than direct viral neuroinvasion. In acute COVID-19, SARS-CoV-2 can enter the CNS and PNS via hematogenous or transsynaptic pathways, facilitated by the angiotensin-converting enzyme 2 (ACE2) receptor, which is expressed on neurons, astrocytes, and vascular endothelial cells, and to a lesser extent on skeletal muscle cells (Perez-Valera et al., 2021[65]; Wang et al., 2020[95]). In the context of long COVID, however, direct viral effects are thought to play a subordinate role, as viral RNA is rarely detected in cerebrospinal fluid (CSF) or peripheral nerve or muscle tissues of symptomatic patients (Matschke et al., 2020[49]). 

Although hypotheses of persisting SARS-CoV-2 reservoirs in tissues contributing to long COVID have been put forth, there is currently a lack of robust neuropathological evidence to support that these reservoirs encompass either the CNS or the PNS (Hejbøl et al., 2022[39]; Swank et al., 2023[85]). Recent evidence indicates that it is highly unlikely that a CNS reservoir of SARS-CoV-2 contributes to neurological long COVID, as studies investigating CSF for SARS-CoV-2 nucleocapsid and spike antigens, have failed to detect evidence of ongoing viral replication in the CSF of patients with neurocognitive deficits and long COVID symptoms. Similarly, although peripheral nerve and skeletal muscle abnormalities, including myopathic changes and amyloid-containing infiltrates, have been documented in individuals suffering from long COVID, viral reservoirs within the PNS have not been reported to date (Appelman et al., 2024[3]; Suh et al., 2021[84]).

On the contrary, viral reservoirs have been identified in histopathological and autopsy studies in other organ systems in long COVID sufferers, including the lungs, skin, breast tissues, and the gastrointestinal tract, along with several blood compartments, encompassing the plasma, granulocytes, and peripheral blood mononuclear cells (Bussani et al., 2023[13]; Goh et al., 2022[34]; Natarajan et al., 2022[56]; Zuo et al., 2024[104]). Interestingly, endotheliopathy leading to increased blood-brain barrier (BBB) permeability has been proposed to comprise a critical pathophysiological link between systemic inflammation and neurological dysfunction in long COVID. For example, a study employing dynamic contrast-enhanced MRI revealed endothelial dysfunction and BBB disruption in a cohort of long COVID patients with brain fog. In addition, the exposure of brain endothelial cells to serum from long COVID patients was found to induce the expression of inflammatory markers, accompanied by an increased adhesion of peripheral blood mononuclear cells to brain endothelial cells *in vitro*. These findings underscore the role of persistent systemic inflammation, endothelial and localized BBB dysfunction in neurological long COVID. In addition, this study suggested that serum biomarkers indicative of BBB breakdown and neuronal injury, including S100β and glial fibrillary acidic protein (GFAP) may hold potential as clinically relevant biomarkers in the near future (Plantone et al., 2024[69]).

In accordance with the viral reservoir hypothesis, the brain-gut axis is also hypothesized to contribute significantly to the neurological manifestations of long COVID. Prolonged shedding of SARS-CoV-2 in the gastrointestinal tract, persisting for several months post-infection, suggests that chronic gastrointestinal infection may perpetuate systemic inflammation, thereby exerting late-effects on the CNS (Gaebler et al., 2021[33]). The persistence of SARS-CoV-2 nucleic acids and proteins in intestinal biopsies in 50 % of long COVID patients suggests that ongoing viral activity could influence not only direct neuro-inflammatory but also secondary neuro-degenerative processes (Gaebler et al., 2021[33]).

**Figure 3:**
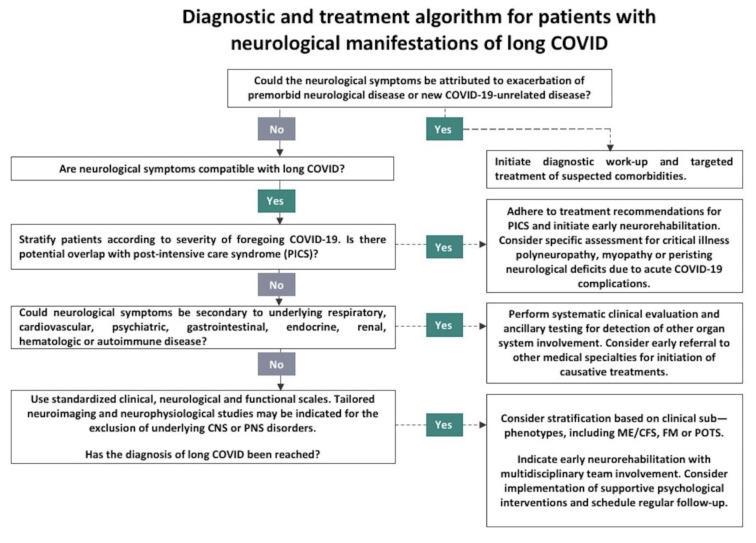
Proposed diagnostic and therapeutic algorithm for patients presenting with neurological manifestations of long COVID. PICS: post intensive care unit syndrome; ME/CFS: myalgic encephalomyelitis/chronic fatigue syndrome; FM: fibromyalgia; POTS: postural orthostatic tachycardia syndrome.

Neuroinflammation appears to comprise a central component of long COVID's pathophysiology (Yang et al., 2021[98]). Persistent activation of the immune system, including abnormal humoral and cellular responses - possibly mediated by mechanisms related to molecular mimicry - contributes to ongoing inflammatory processes (Rojas et al., 2023[74]). To date, elevated levels of pro-inflammatory cytokines, such as interleukin-6 (IL-6), tumor necrosis factor-alpha (TNF-α) and interferon-gamma (IFN-γ), have been implicated in both systemic and neurological manifestations of long COVID (Figure 3(Fig. 3)), albeit inconsistently (Doykov et al., 2020[26]; Yang et al., 2021[98]). Furthermore, auto-antibodies targeting cellular receptors, which have been observed in long COVID patients, are considered instrumental in propagating neuroinflammatory processes (Wallukat et al., 2021[94]). Crucially, reactivation of latent pathogens has also been implicated in the pathogenesis of long COVID, including reactivation of herpesviruses such as Epstein-Barr virus (EBV) and human herpesvirus 6 (HHV-6) (Davis et al., 2023[24]). 

Metabolic dysfunction is another salient feature of long COVID. On the macro level, neuroimaging studies have revealed significant hypometabolism in several brain regions in long COVID patients, including the olfactory bulb, hippocampus, and brainstem - areas with a high density of ACE2 receptors (Chen et al., 2021[19]). Although neuroimaging studies using 18F-FDG PET have not revealed active inflammation in the CNS, ongoing BBB dysfunction and a prolonged hypercoagulable state have been suggested to predispose to hypoxic-ischemic neuronal injury contributing to metabolic derangement and sustained neurological symptoms (Blazhenets et al., 2021[10]; Guedj et al., 2021[37]). Notably, the observed patterns of brain hypometabolism have been shown to extend from the olfactory gyrus to the temporal lobe, hypothalamus, thalamus, brainstem, and cerebellum - regions integral to cognitive functions, sleep, mood, and autonomic regulation (Guedj et al., 2021[37]). On the micro level, impaired cerebral autoregulation, mitochondrial dysfunction, and oxidative stress have all been linked to hypometabolism and unabating neurological symptoms (Davis et al., 2023[24]). 

Autonomic dysfunction merits mention as another distinct facet of long COVID. Dysregulation of heart rate variability (HRV) has been documented in long COVID patients, suggesting impaired autonomic control possibly due to brainstem involvement (Barizien et al., 2021[8]). Besides central dysautonomia, the afferent and efferent pathways of the vagus and glossopharyngeal nerves, both of which are affected in acute SARS-CoV-2 infection, may undergo demyelination or Wallerian degeneration, resulting in sustained autonomic dysfunction (Papadopoulou et al., 2022[62], 2023[61]). In fact, the similarities between long COVID and other post-viral syndromes, such as ME/CFS, warrant consideration. Both conditions share common features, including profound fatigue, post-exertional malaise, dysautonomia and cognitive impairment - symptoms often associated with mitochondrial dysfunction, vascular abnormalities, and neuroinflammation. A notable proportion of long COVID patients also meet the diagnostic criteria for ME/CFS, suggesting that SARS-CoV-2 may trigger similar pathophysiological processes akin to those induced by other pathogens known to cause ME/CFS (Davido et al., 2020[22]). 

Finally, the extent to which genetic susceptibility increases the risk for long COVID remains to date uncertain, despite multiple genetic factors being implicated in its development. For example, variants in the OAS1 gene, part of the oligoadenylate synthetase family, have been linked to prolonged viral clearance and heightened immune responses, potentially precipitating long COVID (Banday et al., 2022[7]). Additionally, mutations in the TLR4 and TLR7 genes, which are instrumental for virus recognition and immunogenicity, have been correlated with severe manifestations of acute COVID-19 and long COVID (Naushad et al., 2023[57]). Additionally, variants in the HLA genes, crucial for immune regulation, alongside mutations in the CCR5 gene, which is primarily involved in immune cell trafficking, are also suggested to increase the susceptibility to long COVID (Hoseinnezhad et al., 2024[41]). Genetic links have also been proposed for the FOXP4 gene, which is predominantly expressed in immune cells and endothelial lung cells, and the ACE2 gene, known as the receptor facilitating SARS-CoV-2 cell entry (Luo et al., 2023[47]; Taylor et al., 2023[88]). However, other studies have failed to replicate the previous findings. Further research is currently underway to uncover these genetic associations, with the aim of facilitating the development of tailored therapeutic strategies that consider genetic predisposition to long COVID.

In parallel, emerging evidence indicates that COVID-19 induces epigenetic changes with significant implications for long-term neurological outcomes. Recent studies have identified alterations in DNA methylation at 42 CpG sites in individuals recovering from COVID-19, suggesting dysregulation of gene expression linked to immune response and metabolic processes (Figure 3(Fig. 3)) (Calzari et al., 2024[14]). Additionally, an increase in stochastic epigenetic mutations (SEMs) has been observed, reflecting genomic instability, alongside accelerated biological aging in patients with long COVID symptoms (Calzari et al., 2024[14]). Importantly, these epigenetic changes may be influenced by specific genetic factors, indicating the possibility of genetically dependent epigenetic alterations. Such modifications could contribute to the persistence of neurological symptoms associated with long COVID, including fatigue and cognitive dysfunction (Shekhar Patil et al., 2024[77]). These findings highlight the need for further research to clarify the underlying mechanisms of long COVID and to develop targeted therapeutic interventions for affected individuals.

## Diagnostic Approach to Patients with Neurological Manifestations of Long COVID

In the absence of widely accepted guidelines specifically tailored to patients with neurological manifestations of long COVID, symptoms that persist beyond the resolution of acute SARS-CoV-2 infection and do not revert to a premorbid baseline are currently regarded as long-term effects of the disease (Stefanou et al., 2022[82]). As an initial step, evaluation for pre-existing or underlying neurological disorders, along with exclusion of other systemic disorders (i.e., long COVID mimics) is crucial before attributing neurological symptoms to long COVID. According to current definitions (Table 1(Tab. 1)), long COVID is defined as “an infection-associated chronic condition that occurs after SARS-CoV-2 infection and is present for at least 3 months as a continuous, relapsing and remitting, or progressive disease state that affects one or more organ systems” (Ely et al., 2024[30]). Crucially, neurological symptoms have not been explicitly defined in the context of long COVID, while current definitions have adopted an inclusive approach, that permits diagnosis even in the presence of “single or multiple” neurological symptoms, or diagnosable conditions.

Despite several criticisms regarding the specificity of the recently proposed NASEM definition, a crucial aspect for clinical practice remains that diagnosis is established purely on clinical grounds, given the absence of reliable biomarkers (Ely et al., 2024[30]). It should also be noted that neither a confirmed history of COVID-19 nor serological evidence of prior SARS-CoV-2 infection is prerequisite for diagnosing long COVID. This is due to the significant number of SARS-CoV-2 infections that either remain asymptomatic or undiagnosed (Zhao et al., 2020[102]). Furthermore, the seroprevalence rates during the post-COVID period show considerable variability and are further complicated by the serological responses induced by SARS-CoV-2 vaccinations (Krammer et al., 2021[43]).

**Figure 4:**
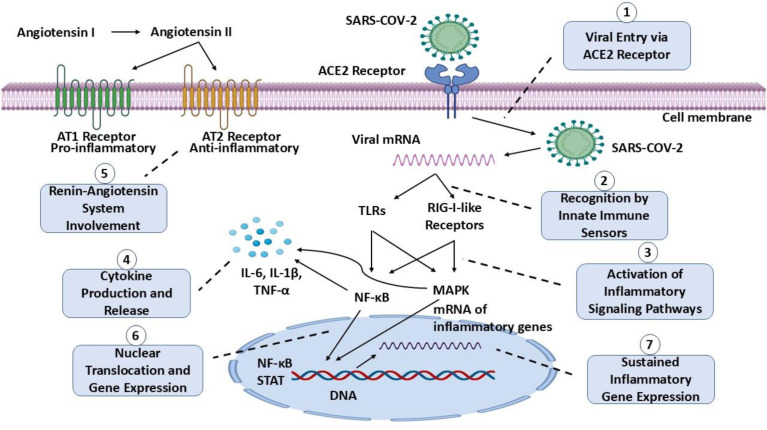
Activation of neuroinflammatory pathways induced by SARS-CoV-2 viral infection. This figure illustrates the sequential mechanisms involved in neuroinflammation following viral entry into host cells, highlighting the immune and inflammatory signaling cascades activated. 1. Viral Entry via ACE2 Receptor: The SARS-CoV-2 viral particle binds to the angiotensin-converting enzyme 2 (ACE2) receptor on the cell surface, allowing viral RNA to enter the host cell and initiate infection. 2. Recognition by Innate Immune Sensors: Viral RNA within the cell is detected by pattern recognition receptors (PRRs), such as Toll-like receptors (TLRs) and RIG-I-like receptors, which recognize viral genetic material and activate innate immune responses. 3. Activation of Inflammatory Signaling Pathways: The binding of viral RNA to PRRs initiates signaling cascades, including the NF-κB and MAPK pathways, which are critical for the host's inflammatory response. These pathways ultimately lead to the production of inflammatory mediators. 4. Cytokine Production and Release: The activated NF-κB and MAPK pathways drive the production and release of pro-inflammatory cytokines, including IL-6, IL-1β, and TNF-α. These cytokines promote local and systemic inflammation, and their upregulation is characteristic of the cytokine storm. 5. Renin-Angiotensin System (RAS) Involvement: Viral binding to ACE2 reduces its normal function, causing an imbalance in the RAS. Angiotensin II, acting primarily through the AT1 receptor, promotes inflammation and oxidative stress. In contrast, the AT2 receptor counteracts these effects by exhibiting anti-inflammatory actions. 6. Nuclear Translocation and Gene Expression: Activated transcriptional factors, including NF-κB and STAT, translocate to the nucleus, where they bind to DNA and initiate transcription of inflammatory genes. 7. Sustained Inflammatory Gene Expression: The continuous expression of inflammatory genes contributes to chronic inflammation, potentially leading to neurological damage and exacerbating disease severity in cases of severe infection. This image was created with BioRender (https://biorender.com). ACE2: Angiotensin-Converting Enzyme 2; AT1: Angiotensin II Type 1 Receptor; AT2: Angiotensin II Type 2 Receptor; IL-6: Interleukin-6; IL-1β: Interleukin-1 beta; MAPK: Mitogen-Activated Protein Kinase; NF-κB: Nuclear Factor kappa-light-chain-enhancer of activated B cells; PRR: Pattern Recognition Receptor; RAS: Renin-Angiotensin System; STAT: Signal Transducer and Activator of Transcription; TLR: Toll-Like Receptor; TNF-α: Tumor Necrosis Factor-alpha

In a previous work conducted by our group, we proposed a detailed algorithm for the clinical approach to patients with neurological manifestations of long COVID (Stefanou et al., 2022[82]). In its latest definition, NASEM emphasized on the similarities between long COVID and other infection-associated chronic conditions, such as ME/CFS (Ely et al., 2024[30]). Notably, very recent research has also shown that sub-phenotypes of long COVID (i.e., including ME/CFS) are steadily emerging, based on data-driven and machine-learning approaches; advancements that we have now incorporated into an updated diagnostic framework (Figure 4(Fig. 4)). For example, distinct temporal patterns have been proposed for neurological sub-phenotypes. We, along with others, argue that neurological long COVID sequelae, which often bear strong clinical resemblance to or fulfill the diagnostic criteria for ME/CFS, FM, PICS, and POTS, could benefit from clinical sub-phenotyping, thereby facilitating optimal diagnostic and therapeutic approaches (Dagliati et al., 2023[21]; Yong and Liu, 2022[100]).

Crucially, clinical practice guidelines, such as those issued by the European Society of Clinical Microbiology and Infectious Diseases (ESCMID), propose symptom-oriented diagnostic and therapeutic approaches for long COVID patients (Yelin et al., 2022[99]). Additionally, other consensus guidelines recommend specific work-up and management strategies for patients presenting with specific neurological symptoms, including fatigue, arthralgia or myalgia, headache, cognitive impairment or brain fog, sleep disorders, anxiety or depression, POTS, dysphagia, olfactory or gustatory disorders, and post-exertional malaise (Seo et al., 2024[76]; Yelin et al., 2022[99]). We should also draw attention to the fact that, although a substantial number of diverse clinical outcome assessment tools have been employed in long COVID research to date, a recent international Delphi consensus study has managed to identify 19 core outcome measurement instruments for clinical practice and long COVID research. Nevertheless, although several instruments were suggested for assessing neurological outcomes - including fatigue, pain, post-exertional malaise symptoms, work or occupational impairment, cognitive and mental health - consensus was not achieved (Gorst et al., 2023[36]). These disparities in clinical practice are also reflected in studies, that highlight the major limitations in capturing long COVID by use of ICD-10 codes (ICD-10: U09.9). In addition, mounting evidence highlights the pressing need for refining clinical definitions and standardizing diagnostic approaches (Henderson et al., 2024[40]; Pfaff et al., 2023[67]). 

## Therapeutic Approach to Patients with Neurological Manifestations of Long COVID

To date, managing neurological symptoms in long COVID patients presents significant challenges for clinicians worldwide and is increasingly practiced in interdisciplinary long COVID clinics across the globe. As previously discussed, the heterogeneity of clinical manifestations necessitates individualized approaches, a fact that, in conjunction with the aforementioned limitations of long COVID definitions, hinders the development of 'one-size-fits-all' recommendations. To date, the therapeutic approach to long COVID has primarily focused on non-pharmacological therapies due to the limited evidence from RCTs supporting the effectiveness of pharmacological approaches, especially in alleviating neurological symptoms in long COVID sufferers (Chee et al., 2023[18]).

Among the pharmacological agents investigated in the context of so-far published RCTs, several have shown promising results; yet, were significantly limited by small sample sizes, heterogeneous populations, selection or other methodological biases, or their results have not been replicated to date by subsequent studies (Table 3(Tab. 3); References in Table 3: Bramante et al., 2023[11]; Harandi et al., 2024[38]; Lau et al., 2024[45]; Momtazmanesh et al., 2023[54]; Pooladgar et al., 2023[70]; Rathi et al., 2021[72]; Tosato et al., 2022[89]). For example, amantadine was associated with significant fatigue-relieving effects in an open-label RCT including 66 patients with fatigue within 30 to 60 days from SARS-CoV-2 infection (33 allocated to the treatment arm receiving 100 mg orally twice daily for two weeks vs. 33 in the control arm receiving no treatment) (Harandi et al., 2024[38]). However, this RCT had significant methodological limitations: (i) it was not double-blinded; (ii) no placebo was used for the control group; and (iii) there were statistically significant between-group differences in demographics, including patient age and body mass index. In another small RCT (comprising 25 patients; 10 in the treatment and 15 in the control arm, respectively), donepezil (5 mg once daily) was found to exert positive effects on specific memory subtests in long COVID patients with cognitive impairment (Pooladgar et al., 2023[70]). Nevertheless, no significant overall improvement in memory scales was recorded over time, with significant limitations including the small sample size, a short follow-up period of 12 weeks, and a single-blind design. Another randomized, double-blind, placebo-controlled trial demonstrated that famotidine (40 mg twice daily), a selective histamine H2 receptor antagonist, was both safe and effective in improving cognitive impairment, depression, and anxiety in long COVID patients (Momtazmanesh et al., 2023[54]). Nevertheless, this trial also had a limited sample size (50 patients; 25 in each group) and brief follow-up (of 12 weeks); thus, its results warrant replication in the context of larger RCTs. In addition, a seminal multicenter, randomized, quadruple-blind, parallel-group, phase 3 trial demonstrated that metformin reduced the incidence of long COVID by 41 % during a 10-month follow-up period compared to placebo. Participants were obese or overweight adults with COVID-19 symptoms for <7 days, who were assigned to receive either metformin plus fluvoxamine, metformin plus placebo, ivermectin plus placebo, fluvoxamine plus placebo, or placebo plus placebo (Bramante et al., 2023[11]). In total, 1126 participants were included (median age, 45; 56 % women, 55 % had received COVID-19 vaccination) who received metformin or matched placebo. The cumulative incidence of long COVID was 10.4 % (placebo) vs. 6.3 % (metformin), a difference that translated into a 41 % reduction with metformin. Notwithstanding the promising findings of this RCT, crucial methodological considerations include: (i) long COVID diagnosis was made at the discretion of a medical provider, rather than by use of pre-defined diagnostic criteria; (ii) selection bias due to the inclusion of individuals who completed the 10-month follow-up; and (iii) limited generalizability due to the exclusion of lower-risk groups. Given the aforementioned limitations, that mainly pertain to the poorly defined long COVID population of this trial, metformin is not routinely recommended for long COVID prevention; nevertheless, future well-designed research is required to shed more light on metformin's preventive and therapeutic potential.

Beyond pharmacotherapy, dietary supplements and vitamins hold a significant role in the therapeutic management of long COVID patients, as demonstrated by findings from recently published RCTs (Table 3(Tab. 3)). In an RCT of 50 long COVID patients, L-arginine and vitamin C supplementation was associated with improvement in physical performance, endothelial function, and fatigue reduction (Pizzorno, 2023[68]). Nonetheless, the study's small, single-center design limits its generalizability, underscoring the need for larger, multi-center trials with extended follow-up to confirm these results and elucidate potential underlying mechanisms. Similarly, the efficacy of the synbiotic SIM01 was investigated in an RCT including 463 long COVID patients (Lau et al., 2024[45]). This trial demonstrated that SIM01 is effective in alleviating neurological symptoms (including fatigue, memory loss, concentration impairment) compared to placebo; albeit with the significant limitation of poorly defined long COVID symptoms, underscored by the absence of standardized global assessment tools. Furthermore, the impact of ImmunoSEB and ProbioSEB CSC3 on long COVID fatigue was evaluated in an RCT comprising 200 patients, with significant improvements in fatigue and quality of life reported after 14 days of intervention compared to placebo, although the study's short duration, lack of psychiatric history data, and absence of long-term follow-up were notable limitations (Rathi et al., 2021[72]).

Building on the insights gained from recent RCTs evaluating pharmacological interventions for long COVID, a complementary body of research has focused on non-pharmacological treatments (Table 4(Tab. 4); References in Table 4: Kuut et al., 2023[44]; McGregor et al., 2024[51]; Santana et al., 2023[75]; Zilberman Itskovich et al., 2022[103]). For example, some benefits have been shown for High Definition transcranial Direct Current Stimulation (HD-tDCS), with an RCT of 70 long COVID patients demonstrating significant reductions in fatigue; though the study's limitations included a lack of adjustment for COVID-19 baseline parameters and absence of Magnetic Resonance Imaging (MRI) to guide HD-tDCS (Santana et al., 2023[75]). In another RCT including 114 long COVID patients (randomly assigned 1:1), Cognitive-Behavioral Therapy (CBT) effectively reduced severe fatigue with benefits lasting up to six months, despite limitations such as lack of blinding and reliance on self-reported outcomes (Kuut et al., 2023[44]). Other approaches have provided some preliminary findings of potential efficacy, such as Hyperbaric Oxygen Therapy (HBOT), which was found in an RCT of 73 patients to be associated with significant improvement in cognitive and psychiatric symptoms, though the small sample size and need for optimization of treatment protocols limit the generalizability of these findings (Zilberman-Itskovich et al., 2022[103]). Additionally, the REGAIN (Rehabilitation Exercise and psycholoGical support After COVID-19 Infection) program, an online, supervised rehabilitation intervention, showed significant improvements in health-related quality of life compared to usual care, albeit with limitations related to blinding and selection/reporting biases (McGregor et al., 2024[51]).

While no consensus has been reached regarding the optimal treatment of long COVID to this day, prevention of long COVID has steadily come into focus. Full vaccination protocols are crucial, based on a growing body of evidence that vaccination significantly reduces the risk of severe illness and subsequent development of long-term sequelae (Seo et al., 2024[76]). Recent research also highlights the necessity of keeping vaccination up to date, including receiving booster doses, to optimize immune protection (Ceban et al., 2023[17]; Watanabe et al., 2023[96]). Simultaneously, early administration of antiviral agents to high-risk individuals diagnosed with COVID-19, in accordance with current clinical practice guidelines, has been demonstrated to diminish disease severity and lower the risk of enduring long COVID sequelae (Seo et al., 2024[76]).

In conclusion, the development of standardized treatment guidelines for managing the neurological manifestations of long COVID remains to date largely elusive. The variability in patient presentations and the evolving landscape of long COVID definitions present substantial obstacles to the implementation of uniform protocols. The diverse symptomatology of long COVID, coupled with inconsistent definitions and diagnostic criteria, complicates efforts to establish comprehensive treatment guidelines. Additionally, many recent RCTs have been constrained by small sample sizes and short follow-up periods, facts that limit the generalizability and long-term applicability of their findings. This ongoing uncertainty highlights the need for further research and the development of comprehensive clinical guidelines to effectively address the complex and diverse manifestations of long COVID.

## Concluding Remarks

This narrative review provides an updated overview of the neurological manifestations of long COVID, illustrating its broader ramifications on a global healthcare and socioeconomic scale. Despite substantial research progress, the absence of a standardized diagnostic framework significantly impedes the development of comprehensive clinical practice guidelines. Currently, the heterogeneity in clinical phenotypes and disease trajectories among patients necessitates multidisciplinary approaches and personalized treatment strategies. Given the significant overlap of long COVID with conditions such as ME/CFS, FM, PICS, and POTS, patients presenting with neurological symptoms may significantly benefit from clinical sub-phenotyping, aiming both to improvement of diagnostic and treatment strategies. 

With respect to the therapeutic landscape, current evidence supports a multimodal approach that integrates both pharmacological and non-pharmacological strategies. Recent RCTs have demonstrated the potential efficacy of various treatments, from immunomodulators and anti-inflammatory agents to extensive neurocognitive and physical rehabilitation programs, advocating for a shift toward an integrative patient management paradigm. However, the limited scale and methodological constraints of these studies restrict the generalizability of the available results, emphasizing the necessity for larger, well-designed RCTs to validate these preliminary findings and expedite the development of robust treatment guidelines. Importantly, while treatment alternatives await validation, prevention of long COVID has come to the foreground, both through vaccination as well as implementation of antivirals in selected high-risk populations. We emphasize that vaccination campaigns should continue unabated, as they are considered integral to reducing the global burden of long COVID, including its long-term neurological manifestations. 

As a concluding remark regarding the current state of epidemiological data, there is congruent evidence that approximately one third of COVID-19 survivors will experience prolonged neurological sequelae that persist for at least 12 months post-infection. Extending the tentative inference of a 10 % incidence of long COVID among the more than 651 million documented COVID-19 cases globally, the estimated cases of neurological manifestations related to long COVID likely exceed 20 million worldwide (Davis et al., 2023[24]). Considering the detrimental effects on individuals' quality of life, as well as the immense repercussions on population health and the incremental productivity losses worldwide, joint international initiatives are warranted to address the current challenges in defining, diagnosing, and managing long COVID (Carlile et al., 2024[15]). Finally, in addressing neurological symptoms, standardized approaches are crucial to allow for better delineation of this chronic post-infectious disorder. Such collaborative strategies are integral to support policymakers in allocating healthcare resources and devising prevention and treatment agendas aimed at mitigating the galloping burden of long COVID globally.

## Figures and Tables

**Table 1:**
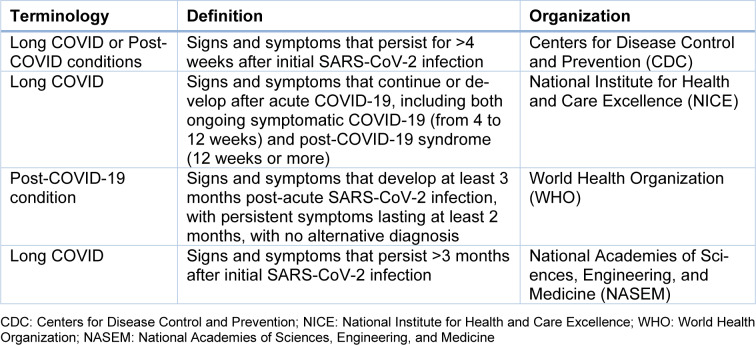
Comparative analysis of terminology and definitions of long COVID (Chou et al., 2024; Ely et al., 2024; Soriano et al., 2022)

**Table 2:**
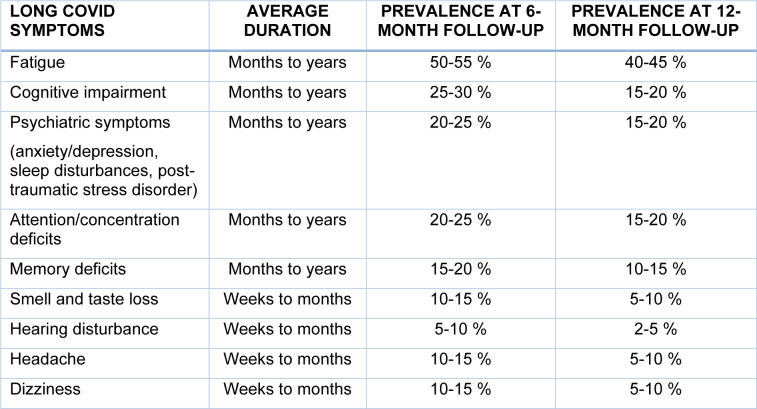
Average duration and prevalence of neurological manifestations of long COVID at 6- and 12-months post-infection (Arnold et al., 2021; Badenoch et al., 2022; Dorobisz et al., 2023; Li et al., 2023; Păunescu et al., 2023; Peter et al., 2022; Rivera-Izquierdo et al., 2022; Tana et al., 2022; Vaira et al., 2020)

**Table 3:**
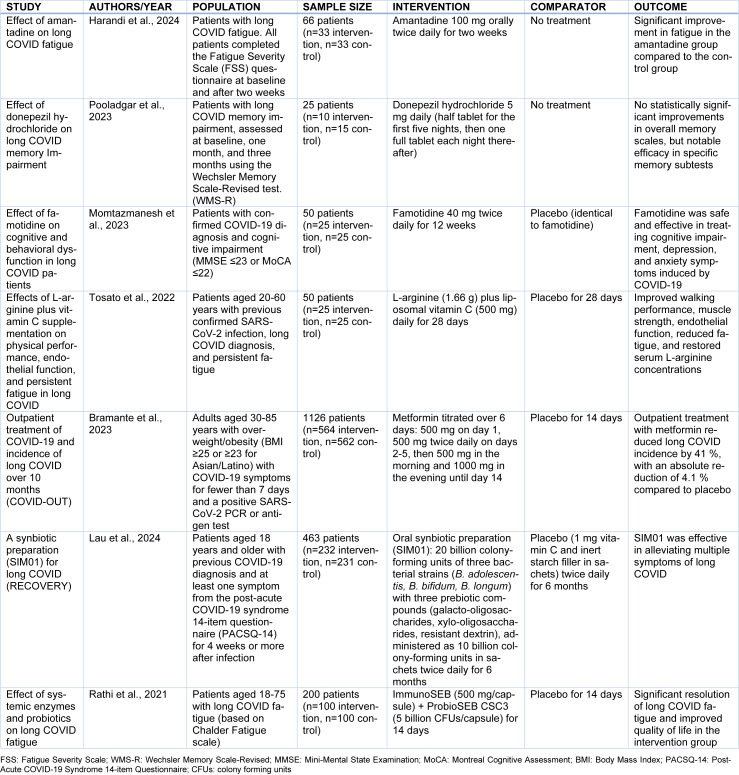
Summary of randomized-controlled clinical trials on pharmacological interventions for neurological manifestations of long COVID

**Table 4:**
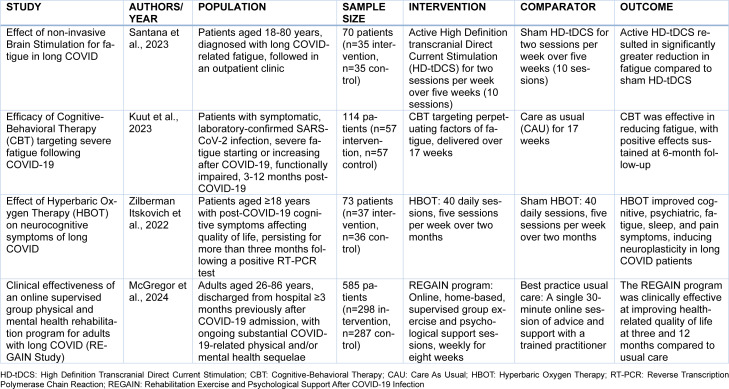
Summary of randomized-controlled clinical trials on non-pharmacological interventions for neurological manifestations of long COVID

**Figure 1:**
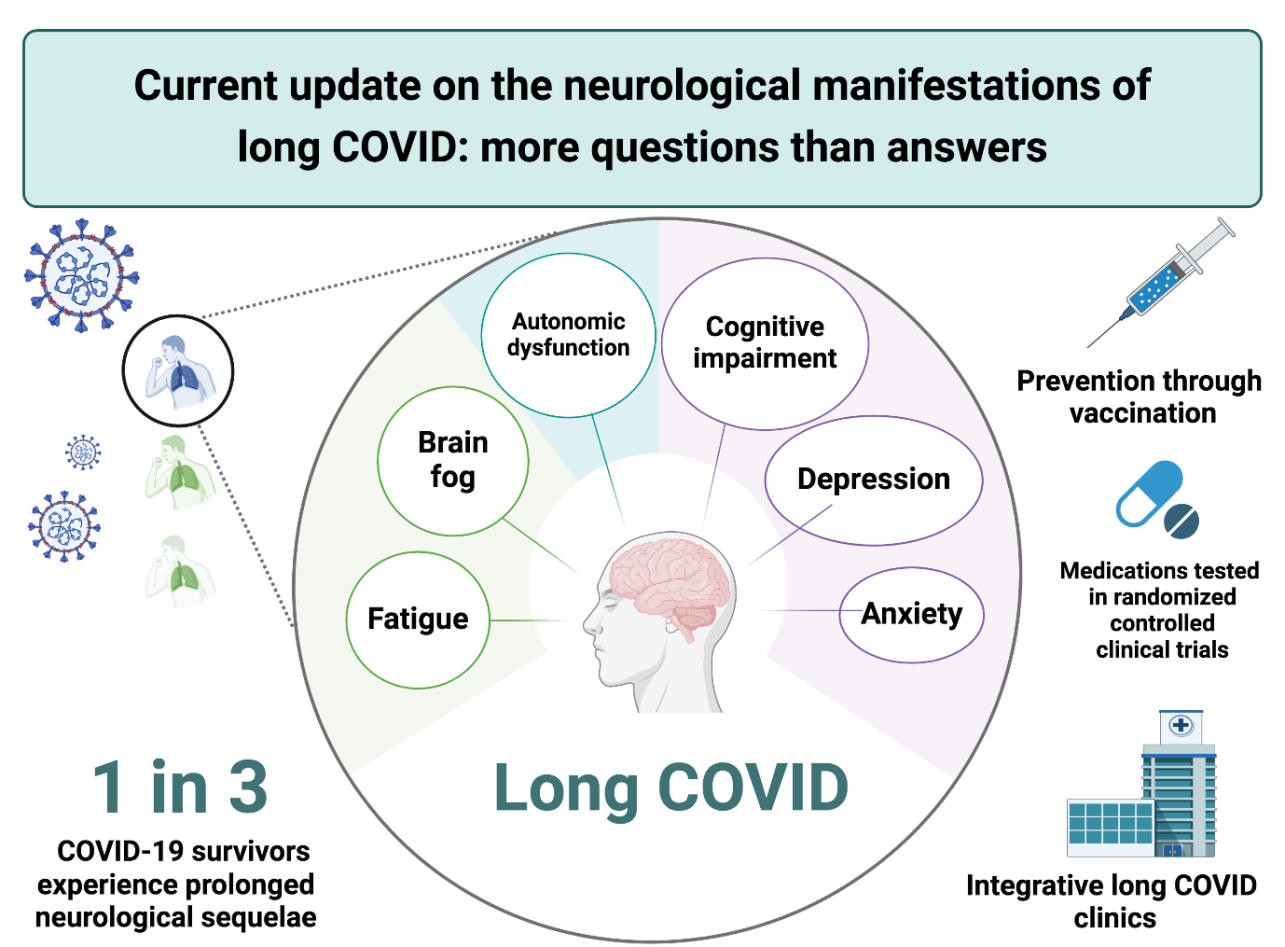
Graphical abstract
